# Relationship between the True Digestibility of Dietary Calcium and Gastrointestinal Microorganisms in Goats

**DOI:** 10.3390/ani10050875

**Published:** 2020-05-18

**Authors:** Yuehui Liu, Ali Mujtaba Shah, Lizhi Wang, Lei Jin, Zhisheng Wang, Bai Xue, Quanhui Peng

**Affiliations:** 1 Institute of Animal Nutrition, Sichuan Agricultural University, Chengdu 611130, Sichuan, China; Liuyuehui013@126.com (Y.L.); alimujtabashah@sbbuvas.edu.pk (A.M.S.); 13551552940@163.com (L.J.); wangzs@sicau.edu.cn (Z.W.); xuebai68@163.com (B.X.); pengquanhui@126.com (Q.P.); 2 Department of Livestock Production, Shaheed Benazir Bhutto University of Veterinary and Animal Science, Sakrand 67210, Pakistan

**Keywords:** goat, true digestibility of calcium, high-throughput sequencing, gastrointestinal tract, bacteria

## Abstract

**Simple Summary:**

The specific enzymes secreted by microorganisms in the gastrointestinal tract (GIT) of ruminants, such as phytase, can catalyze the decomposition of calcium compounds (e.g., phytic acid) and release bound calcium for the absorption of animals. Therefore, we speculate that gastrointestinal microbes could be a factor affecting digestion and absorption of dietary calcium. However, little related research has been reported. In the present study, we found that the true digestibility of calcium (TDC) in goats is related to gastrointestinal bacteria. Some gastro-intestinal bacteria, such as ruminal Prevotella, were beneficial for true host digestibility of dietary calcium.

**Abstract:**

The current study was performed to examine the relationship between the true digestibility of calcium (TDC) in the diet and bacterial community structure in the gastrointestinal tract (GIT) of goats. Twenty-six Nubian healthy female goats were selected as experimental animals, and their TDC was determined using metabolic experiments. Eight goats were grouped into the high digestibility of Calcium (HC) phenotype, and another eight were grouped into the low digestibility of Calcium (LC) phenotype. Their bacterial 16S rRNA gene amplicons from the rumen, abomasum, jejunum, cecum, and colon contents were sequenced using next-generation high-throughput sequencing technology. In the rumen, 239 genera belonging to 23 phyla, 319 genera belonging to 30 phyla in the abomasum, 248 genera belonging to 36 phyla in the jejunum, 248 genera belonging to 25 phyla in the colon and 246 genera belonging to 23 phyla in the cecum were detected. In addition, there was a significant correlation between the TDC and the relative abundance of *Candidatus_Saccharimonas*, *Christensenellaceae*_*R*-7_*group*, *Mogibacterium*, *Prevotella_*1, *Prevotella_UCG*_004, *Ruminococcus*_2, *Saccharibacteria* in the rumen, *Eubacterium_coprostanoligens_group*, *Lachnospiraceae_ND*3007_*group*, *Lachnospiraceae_NK*3*A*20*_group, p-*1088-*a*5_*gut_group*, and *Planctomycetes* in the abomasum, *Butyrivibrio* in the cecum, and *Fibrobacter* in the cecum were observed. This study suggests an association of GIT microbial communities as a factor influencing TDC in goats.

## 1. Introduction

The gastrointestinal tract (GIT) of ruminants contains many anaerobic microorganisms that have a symbiotic relationship with their hosts, such as bacteria, protozoa, and fungi. The feed ingested by ruminants supplies nutrition to these microorganisms, and microbial communities play an important role in promoting the digestion and utilization of dietary nutrients by the host [1]. The rumen is one of the most critical places for ruminants to digest feeds but cannot secrete digestive enzymes. The digestion of feed occurring in the rumen depends entirely on the symbiotic microorganisms [2]. Therefore, a stable rumen micro-ecosystem is necessary for ruminants to digest plant feed. Our recent study shows that microbes in the small intestine and large intestine play an essential role in the digestion and utilization of nutrients as well as rumen, such as nitrogen [3] and phosphorus (unpublished data of our research group).

Calcium (Ca) is the third major nutrient in the ruminant’s diet after energy and protein. Calcium maintains osmotic equilibrium and acid-base balance, and involves in muscle contraction, protein synthesis, transmission of nerve impulses, and other physiological activities. Calcium is the most abundant micro-mineral, and its concentration in the body is 3% to 5% [4]. Correct inclusion of calcium in the ration is imperative for proper physiological function and growth of the ruminants. Concerns for maintaining adequate animal welfare, providing cost-effective rations, and reducing nutrient excretion have driven the advancement in knowledge about the nutrients digestibility, which can refer to the ileal or total tract digestibility (TTD) [5]. TTD is an appropriate measure for determining the digestibility of calcium in feed ingredients, although negligible amounts of digestion occur after the small intestine, and no net secretion of endogenous calcium takes place in the large intestine [6]. TTD can be expressed as apparent total tract digestibility (ATTD) or standardized total tract digestibility (STTD). Because of the existence of endogenous losses of calcium from the GIT, STTD is more accurate than ATTD in evaluating the digestibility of feed ingredients. Thus far, there is little information for the STTD of calcium in goats.

The previous study shows that the digestibility of dietary calcium is mainly affected by the level of dietary calcium [7], the ratio of calcium to phosphorus [8], and the type of feeds [9]. Calcium usually chelates other dietary compounds and can be absorbed as soluble or ionic forms by animals. The specific enzymes secreted by microorganisms in the GIT of ruminants, such as phytase, can catalyze the decomposition of calcium compounds (e.g., phytic acid) and release bound calcium for the absorption of animals [10,11]. Therefore, we speculate that gastrointestinal microbes be a factor affecting digestion and absorption of dietary calcium. However, little related research has been reported. In view of this, the present study was conducted to compare the microbial diversity in the GIT of goats with different true digestibility of calcium (TDC) using 16S rDNA high-throughput sequencing technology and analyzed the correlation between TDC and gastrointestinal microbes.

## 2. Materials and Methods

The experimental protocol used in the present study was approved by the Animal Policy, and Welfare Committee of the Agricultural Research Organization of Sichuan Province, China, and was in accordance with the guidelines of the Animal Care and Ethical Committee of the Sichuan Agricultural University code (Code-SYXK-Chuan-2014-184).

### 2.1. Animals and Sampling

The present experiment was performed at the experimental farm of Animal Nutrition Institute, Sichuan Agricultural University, China. Twenty-six (10 months old) healthy female Nubian goats having similar initial body weight (24.25 ± 2.47 kg) were moved individually into metabolic crates equipped with feeders and water buckets to evaluate nutrient digestibility. During the experiment, all goats were fed a restricted level of (4% body weight, dry matter (DM) total mixed ration in two equal amounts every day at 8:30 a.m. and 5:30 p.m. The composition and nutritive values of the experimental diets are presented in Table 1.

Referring to the previously described process of difference level technique (DLT) [12], we designed two metabolic experiments (stage 1 and stage 2) to measure the true digestibility of dietary calcium. Each metabolic experiment stage (1 and 2) lasted for 20 days, which consisted of an adaptation period (14 days) and a formal metabolism experiment period (six days). The amount of feces, urine, daily feed intake, and residual intake were recorded, and all feces and urine were collected during the six-day metabolism experiment period for the calculation of nutrients digestibility. Blood samples (10 mL) were collected from the jugular vein of each goat into test tubes at, approximately, before morning feeding and at the end of the second metabolism experiment. Serum was obtained after centrifugation (3000× *g*, 15 min) and then stored at −20 °C for later analysis. Then, all goats were slaughtered before morning feeding after two metabolism experiments. Different GITs were separated by a ribbon to prevent the shifting of luminal contents from one site to another; to get representative samples, we mixed contents of each tract respectively, and the samples of the rumen, abomasum, jejunum, cecum, and colon contents were obtained. Eight tubes (50 mL each) of rumen contents (liquid and solid) and four tubes (15 mL each) of abomasum, jejunum, cecum, and colon digesta were collected. Partial of the ruminal samples were subjected to pH evaluation with a portable acidity meter (PHS-100, Tianqi Mdt InfoTech, Ltd., ShangHai, China) [13], and strained through four layers of cheesecloth to collect rumen liquid immediately. The rumen liquid (20 mL) was fixed with a meta-phosphoric acid solution (5 mL, 250 g/L) and kept in refrigerating cabinet (−20 °C) for later measurement of the volatile fatty acid (VFA; acetate, propionate, and butyrate) concentration. The remaining rumen and other gastrointestinal segment samples were kept at −80 °C for the analysis of bacterial composition and structure.

### 2.2. Analysis of Samples and Grouping

All the feed and feces were dried at 65 °C for 96 h in a forced-air oven, and then samples were ground to pass through a 1 mm sieve. All feed and fecal samples were analyzed for dry matter (DM), ash, ether extract (EE), crude protein (CP), Neutral detergent fiber (NDF) and acid detergent fiber (ADF) following the procedures outlined by the Association of Official Analytical Chemists [14]. The concentration of serum phosphorus, calcium, and the activity of serum alkaline phosphatase was determined by an automatic biochemical analyzer (Automatic Analyzer 3100, Hitachi, Tokyo, Japan). The DM was determined on an aliquot sample to establish the residual water content after drying for 24 h at 103 °C, and the ash content was determined after ignition of a weighed sample in a muffle furnace at 550 °C for 6 h. The corresponding analytical result was expressed on a DM basis. For the determination of crude protein (CP) and ether extract (EE), the kjeldahl and Soxhlet extraction methods were used, respectively. The CP was determined as total N × 6.25. Neutral detergent fiber (NDF) and acid detergent fiber (ADF) were measured using the filter bag technique without supplementation of sodium sulphite or heat-stable amylase. The rumen liquid was centrifuged at 4 °C for 10 min (12,000× *g*), and the sediment was discarded. The concentrations of acetate, propionate, and butyrate in the supernatant were determined using gas chromatography (GC-2014FRGA1, Shimadzu, Tokyo, Japan) [15]. The TDC was calculated using the previously described method [12]. The calculation of TDC presented as under:

TDC (%) = (I_II_ − F_II_ + E)/I_II_ × 100) (1)

where I_II_ is the calcium intake of second metabolism experiment, and F_II_ is the fecal calcium of the second metabolic experiment, E is the amount of endogenous calcium and was calculated as follows:

E = [F_II_ × I_I_ − (F_I(tc)_ − F_I(ic)_) × I_II_]/(I_I_ − I_II_) (2)

where I_I_ and I_II_ are the organic calcium intake of the first and the second metabolic experiment, respectively. F_I(tc)_ and F_I(ic)_ are the total calcium and inorganic calcium in the feces of the first metabolic experiment, respectively. F_I(ic)_ was calculated as follows:

F_I(ic)_ = I_I(ic)_ × (1 − TDC_I(ic)_) (3)

where I_I(ic)_ and TDC_I(ic)_ are the inorganic calcium intake and the true digestibility of inorganic calcium of the first 6-day metabolic experiment period, respectively, and TDC_I(ic)_ was calculated according to the principle of DLT [16] as follows:

TDC_I(ic)_(%) = (D_I_ − D_E_)/D_I_ × 100 (4)

where D_I_ and D_E_ are the difference of total calcium intake and fecal total calcium between the first and the second metabolic experiment, respectively.

The mean and standard deviation (SD) of the TDC used to group for selected goats into high digestibility of calcium (HC, TDC > mean + 0.5 × SD) phenotype and low digestibility of calcium (LC, TDC < mean − 0.5 × SD) phenotype, referring to the previously described method [17,18].

### 2.3. DNA Extraction and Amplification

The samples of gastrointestinal content kept at −80 °C were taken out and thawed on ice. Before DNA extraction, the gastrointestinal contents were transferred and rinsed with sterile, phosphate-buffered saline (PBS) (pH 7.0) to the four layers of gauze and then the flushing fluid was collected into another sterile EP tube. Subsequently, the flushing fluid was immediately centrifuged at 10,000× *g*, and the supernatants were removed. The remaining samples were used for DNA extraction. Then, the DNA samples were extracted referring to the method described by Guo [19] using the TIANamp Bacteria DNA Kit (TIANGEN, Peking, China) according to the manufacturer’s guidelines. The quality measurement of the extracted bacterial was performed using agarose electrophoresis with a NanoDrop 8000 spectrophotometer (Thermo Fisher Scientific, Brisbane, Australia).

The high-quality DNA and a pair of bacteria-specific primers (forward primer sequence: 5′-GTGCCAGCMGCCGCGGTAA-3′ (515F), reverse primer sequence: 5′-GGACTACVSGGGTATCTAAT-3′ (806R)) [20] were used to amplify the V4 hypervariable region of the bacterial 16S rRNA gene. A unique 5-8-base error-correcting barcode for each sample was added to the end of the primer 515F, which allowed sample multiplexing during sequencing. The procedures for the amplification were as follows: initiated at 94 °C for 3 min to denature, followed by 30 cycles at 94 °C for 30 s, 58 °C for 30 s and 72 °C for 90 s, and extended at 72 °C for 5 min. The total volume of the reaction mixture was 50 μL, which consisted of 400 nM primer (each primer 200 nM), 5 μL dNTP (2.5 mmol/L) mixture, 5 μL of 10 × Ex Taq buffer (20 mmol/L Mg^2+^, TaKaRa Inc., Dalian, China), 0.35 μg of template DNA, 2 mM MgCl_2_, four units of Taq DNA polymerase (Takara Inc., Dalian, China), and approximately 37 μL milli-Q water. When the amplification procedures were finished, a PCR Clean-Up system (Promega, Madison, USA) with a purification kit (QIAGEN, Victoria, Australia) was used to purify the amplicons, and a QuantiFluor™-ST fluorometer (Promega, Beijing, China) was used to quantify them. Finally, the high-quality amplicons were sent to Novogene Technology Company (Beijing, China) and sequenced on the MiSeq Illumina Sequencing Platform according to the protocols described by Caporaso [21].

### 2.4. Bioinformation Analysis

According to the previously described method, the acquired reads from Novogene was analyzed using QIIME pipeline software (version 1.8.0) [22]. The sequences containing uncertain nucleotides, unmatched barcode, and three continuous nucleotides with Q values less than 20 were discarded, and then based on the Uchime algorithm implemented in QIIME, the chimeric sequences were eliminated using Usearch V7.0 [23,24]. A pre-clustering methodology was used to reduce sequencing noise [25]. Finally, the resultant clean and high-quality sequences were clustered into operational taxonomic units (OTUs), and the representative sequence of each OTU was chosen using Uclust method at 97% similarity [26], following bysingletons removed by Uchime. The representative of each OTU was aligned against the Greengenes database (http://greengenes.lbl.gov) and assigned to taxonomy using RDP Classifier [27]. Seven alpha diversity indices of the bacterial communities (Observed_species, Shannon, Simpson, Chao1, ACE, Goods_Coverage, and PD_whole_tree) were calculated at a depth of 21,854 sequences. Beta diversity was visualized using principal coordinate analysis (PCoA), as measured using an unweighted UniFrac distance matrix [28]. The bacterial compositions at phylum and genus level were presented using relative abundance histograms drawn by the OriginPro software (version 9.0). In addition, a heatmap created by R version 3.4.2 software program, was used to show the genera shared by all samples. All sequence data obtained in this study were deposited in the Sequence Read Archive (SRA) of the NCBI database (accession number: SRP185613).

### 2.5. Statistical Analysis

The difference between the HC and LC groups, the relative bacterial abundance, was analyzed by a nonparametric test of two samples, and other parameters were analyzed by unpaired *t*-test using SPSS Statistics software v. 19.0 (IBM, Armonk, NY, USA), which was also used to perform the Spearman rank correlation analysis between the relative abundance of bacteria and TDC. The results were presented as the means ± SD, and the significant and extremely significant levels were set at *p* < 0.05 and *p* < 0.01, respectively.

## 3. Results

### 3.1. The TDC of Goats

The TDC of 26 goats varies from 53.25% to 80.57%, with an average of 68.36% ± 8.81%. The TDC in HC group is 76.93% ± 4.12%, and 59.62% ± 2.91% in the LC group, the difference in TDC between the HC and LC animals was significant (*p* < 0.05).

### 3.2. Plasma Biochemical, Ruminal Fermentation and Nutrient Apparent Digestibility Indices

In the present study, we found no significant difference in serum calcium content between the HC and LC group (*p* > 0.05). Additionally, there was no significant difference in rumen pH and the proportion of volatile fatty acids. Besides, the apparent digestibility of basic nutrients had no difference between the groups either (*p* > 0.05) (Table 2).

### 3.3. Data Acquired from Sequencing

**Figure 1:**
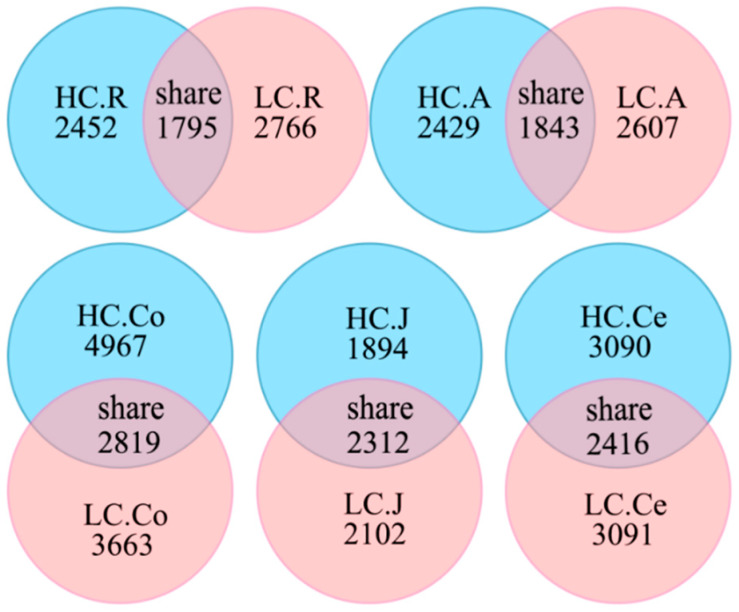
Venn diagram representation of the shared and exclusive OTUs. Note: HC.R: rumen of High Digestibility of Calcium group (HC); LC.R: rumen of Low Digestibility of Calcium group (LC); HC.A: abomasum of HC; LC.A: abomasum of LC; HC.J: jejunum of HC; LC.J: jejunum of LC; HC.Co: colon of HC; LC.Co: colon of LC; HC.Ce: cecum of HC; LC.Ce: cecum of LC.

**Figure 2:**
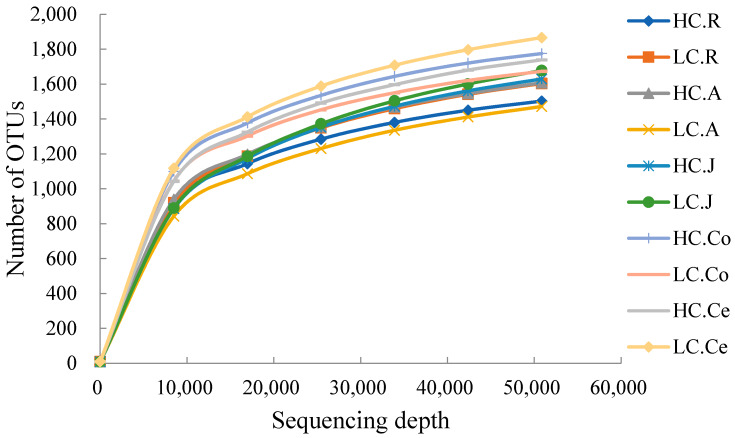
Rarefaction curves of each group. Note: HC.R: rumen of High Digestibility of Calcium group (HC); LC.R: rumen of Low Digestibility of Calcium group (LC); HC.A: abomasum of HC; LC.A: abomasum of LC; HC.J: jejunum of HC; LC.J: jejunum of LC; HC.Co: colon of HC; LC.Co: colon of LC; HC.Ce: cecum of HC; LC.Ce: cecum of LC.

In the sequencing analysis of 16S rDNA, a total of 5,711,204 high-quality sequences were generated, with 1,224,443 in the rumen, 1,227,513 in the abomasum, 1,191,390 in the jejunum, 1,200,863 in the colon, and 1,087,964 in the cecum. With an average of 76,527 ± 4781 per sample in the rumen, 76,719 ± 5609 in the abomasum, 74,461 ± 6012 in the jejunum, 75,053 ± 4667 in the colon, and 72,530 ± 7954 in the cecum. At a 97% nucleotide sequence identity between reads, 7013 OTUs were detected in the rumen, 6879 in the abomasum, 6308 in the jejunum, 11,449 in the colon and 8597 in the cecum, with an average of 2431 ± 153 and 2383 ± 226 per sample in the rumen of the HC and LC groups, 2086 ± 186 and 2178 ± 105 in the abomasum, 2313 ± 179 and 2085 ± 271 in the jejunum, 3052 ± 374 and 2848 ± 255 in the colon, and 2609 ± 358 and 2948 ± 163 in the cecum, respectively. The numbers of shared OTUs between groups were presented in Figure 1, with 1795 in the rumen, 1843 in the abomasum, 2312 in the jejunum, 2819 in the colon, and 2416 in the cecum. A rarefaction curve analysis for the OTUs (Figure 2) indicated that sequencing captured the majority of bacteria across the GIT.

### 3.4. Alpha Diversity and Beta Diversity Analysis

We evaluated the richness and evenness of the microbiota across the GIT by the alpha diversity indices. Table 3 shows that most alpha diversity indices were not significantly different (*p* > 0.05) between groups. However, the observed_species and PD_Whole_Tree in the abomasum of HC were significantly (*p* < 0.05) higher than those of LC, and the goods_coverage of cecum were significantly higher than that of LC.

**Figure 3:**
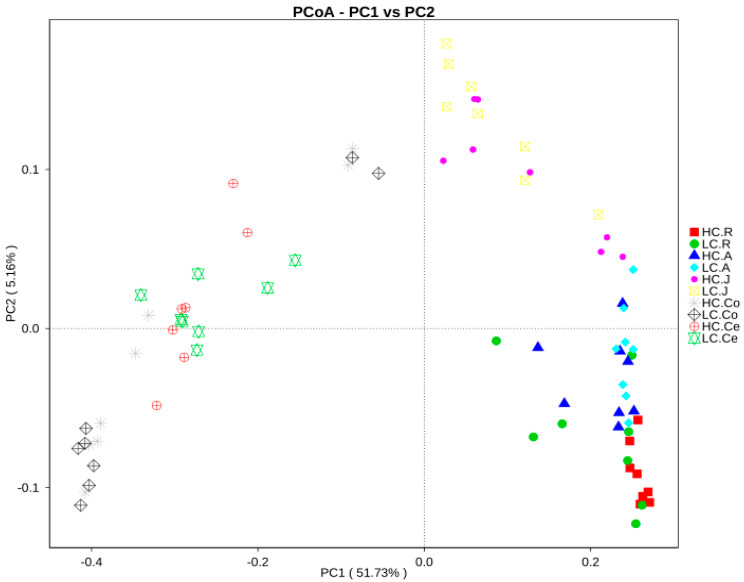
Principal coordinate analysis of bacterial samples from the gastrointestinal tract. A greater distance between two samples indicated a lower similarity. The percentage of variation explained by PC1 and PC2 are indicated on the axis. Note: HC.R: rumen of High Digestibility of Calcium group (HC); LC.R: rumen of Low Digestibility of Calcium group (LC); HC.A: abomasum of HC; LC.A: abomasum of LC; HC.J: jejunum of HC; LC.J: jejunum of LC; HC.Co: colon of HC; LC.Co: colon of LC; HC.Ce: cecum of HC; LC.Ce: cecum of LC.

Overall, one picture of the microbial composition of the samples in group HC and LC was obtained by PCoA, based on the relative abundance profiles of bacterial taxa. As shown in Figure 3, a closer distance between two points indicated a greater similarity of the samples, and the percentage of variation was elucidated by PC1 and PC2 as indicated by the axis. In the present study, PC1 explained 51.73% of the variation, and PC2 explained 5.16%. In summary, the analysis showed that microbial communities from the same/adjacent GIT regions (rumen, jejunum and large intestine) were more similar to each other than to those from other regions (Figure 3).

### 3.5. Bacterial Composition of the GIT

**Figure 4:**
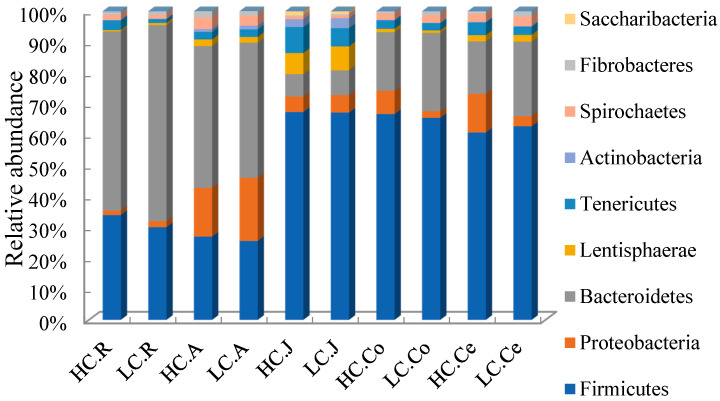
Bacterial compositions across the gastrointestinal tract at the phylum level (only the top 10 abundant phyla are presented). Note: HC.R: rumen of High Digestibility of Calcium group (HC); LC.R: rumen of Low Digestibility of Calcium group (LC); HC.A: abomasum of HC; LC.A: abomasum of LC; HC.J: jejunum of HC; LC.J: jejunum of LC; HC.Co: colon of HC; LC.Co: colon of LC; HC.Ce: cecum of HC; LC.Ce: cecum of LC.

At the phylum level, 23 taxa were identified in the rumen and cecum, 30 in the abomasum, 36 in the jejunum, and 25 in the colon. Data of top 10 microorganism populations were analyzed (Figure 4). The dominant bacteria in the rumen and abomasum were similar, the most abundant phylum in the rumen and abomasum was Bacteroidetes (HC 56.73%, LC 62.31% in the Rumen; HC 44.79%, LC 43.11% in the abomasum), and the secondary phylum was Firmicutes (HC 33.65%, LC 29.91% in the rumen; HC 26.80%, LC 25.63% in the abomasum). While in the gut, the dominant bacteria were Firmicutes (HC 63.73%, LC 63.75% in the jejunum; HC 64.28%, LC 63.27% in the colon; HC 59.26%, LC 60.55% in the cecum).

**Figure 5:**
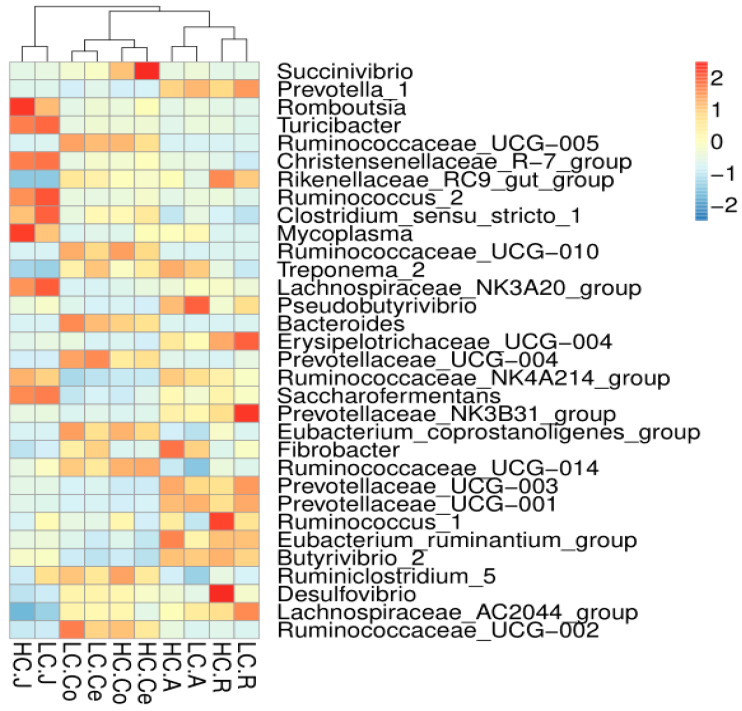
Heatmap of the core genera across the gastrointestinal tract (the relative abundance of microbes was log-transformed, and only the top 32 abundant genera are presented). The closer to the color red, the higher the relative abundance, while the closer to the color blue, the lower the relative abundance.

A total of 239 genera were detected in the rumen, 319 in the abomasum, 248 in the jejunum, 248 in the colon and 246 in the cecum. The average relative abundances of the shared genera are displayed in a heatmap (Figure 5). Then, the top ten microorganism populations were analyzed at genus level. As shown in Figure 5, the dominant genera in the rumen and abomasum was *Prevotella_1* (HC 12.91%, LC 17.97%), and the second dominant genera in the rumen was *Rikenellaceae_RC9_gut_group* (HC 11.37%, LC 9.20%), while *Succinivibrionaceae_UCG-002* in the abomasum (HC 9.37%, LC 13.57%). The difference is, in the jejunum, the most two abundant genera were *Romboutsia* (HC 13.43%, 8.24%) and *Christensenellaceae_R-7_group* (HC 8.86%, 9.11%). In the colon and cecum, the dominant genera were *Ruminococcaceae_UCG-005* (HC 11.30%, LC 12.43% in the colon; HC 9.02%, LC 11.15% in the cecum), Succinivibrio (HC 6.35%, LC 1.19% in the colon; HC 11.37%, LC 1.36% in the cecum) and Rikenellaceae_RC9_gut_group (HC 5.74%, LC 7.99% in the colon; HC 5.37%, LC 7.00% in the cecum) were the primary genus both in the colon and cecum.

### 3.6. Comparisons of Bacterial Composition between HC and LC Group

Compared with the relative abundance of bacteria in phylum to genus level between the HC and LC groups, significant differences were found (Table 4). At the phylum level, the relative abundance of Chloroflexi and Planctomycetes in the abomasum were significantly higher (*p* < 0.05), while Lentisphaerae in the rumen, Bacteroidetes, and Fibrobacteres in the colon were significantly lower (*p* < 0.05) in the HC compared with LC group. At genus level, in the rumen, the relative abundance of *Christensenellaceae_R-7_group*, *Ruminococcus_2*, *Quinella*, *Ruminococcaceae_UCG-004*, *Mogibacterium*, *Family_XIII_UCG-002*, *Candidatus_Saccharimonas*, *Ruminococcaceae_UCG-007* in the HC group were significant higher (*p* < 0.05) than that in the LC group, while *Prevotella_1*, *Prevotellaceae_UCG-003*, *Pseudobutyrivibrio*, *Prevotellaceae_UCG-004*, *Succinivibrionaceae_UCG-002,* and *Bacteroides* were significant lower (*p* < 0.05) in the HC group. In the abomasum, the relative abundance of *Rikenellaceae_RC9_gut_group*, *Succiniclasticum*, *Ruminococcus_1*, *Succinivibrio*, *Lachnospiraceae_ND3007_group*, *Ruminococcaceae_UCG-010*, *Eubacterium_coprostanoligenes_group*, *Ruminococcaceae_UCG-004,* and *p-1088-a5_gut_group* were significantly higher in the HC group compared to the LC group. On the contrary, *Lachnospiraceae_NK3A20_group*, *Ruminococcaceae_UCG-014*, *Acetitomaculum,* and *Prevotellaceae_YAB2003_group* were significantly lower (*p* < 0.05) than that in the LC group. Furthermore, *Bacteroides* and *Fibrobacter* in the colon, *Ruminiclostridium_1,* and *Butyrivibrio* in the cecum were significantly lower (*p* < 0.05) in the HC group compared to the LC group.

### 3.7. Correlation between the TDC and the Bacterial Composition across the GIT

**Figure 6:**
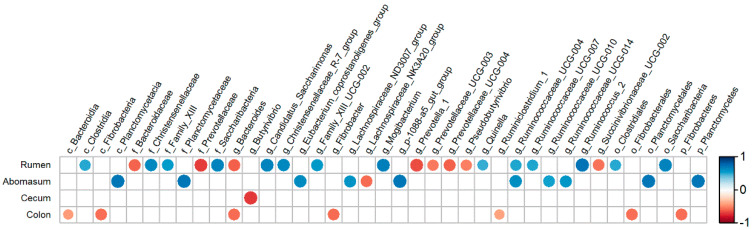
Correlation analysis between the bacteria across the gastrointestinal tract and the true digestibility of calcium in goats. Correlations are indicated by large circles and weaker correlations by small circles. The scale colors denote whether the correlation is positive (closer to 1, red circles) or negative (closer to −1, blue circles).

The correlation between the TDC and the bacterial composition across the GIT are presented as correlation coefficient diagram (Figure 6). The results showed that in the rumen, one class, two orders, three families, and seven genera were positively correlated with the TDC; on the contrary, two families and six genera were negatively correlated with TDC. In the abomasum, one phylum, one class, one order, one family, and six genera had a significant positive correlation with the TDC. The only *Lachnospiraceae_NK3A20_group* was negatively correlated with the TDC. Otherwise, no bacteria with a strong correlation with the TDC were found in the jejunum. And in the colon, TDC was negatively involved in the relative abundance of one phylum, one class, one order, and two genera. Moreover, only one genus (*Butyrivibrio*) had a significant negative correlation with the TDC in the cecum.

## 4. Discussion

In the present study, the TDCs and the standardized total tract digestibility of calcium of the goats were determined using two metabolic experiments, and their mean was 68.36%. Martz [29] reported that the true digestibility of calcium from corn silage in nonlactating dairy cows was 34.4% and 43.7% for high and low calcium rations, respectively. González-Vega [30] reported that the STTD of calcium from canola in growing pigs was 47.93%. Liu [31] found that the standardized availability values of calcium in soybean meal fluctuated between 51.14% and 41.49% for broiler chicks. It was evident that the digestibility of calcium in plant-origin feed is higher in goats compared with monogastric animals and dairy cows.

Historically, the recommended amount of calcium requirement in ruminants have been expressed on a total calcium basis. Although inorganic calcium supplements are relatively cheap, accurate formulation of calcium is essential, because adding calcium above the requirement is detrimental to animal performance and metabolism of other nutrients; therefore, it is necessary to determine not only the digestibility of feed ingredients but also the factors that may affect the digestion of calcium. The digestibility of calcium was mainly influenced by the level of dietary calcium [7], the ratio of Calcium to phosphorus [8], and origin of feed ingredient [9]. However, the current study indicated that the gastrointestinal bacteria might be a factor effecting the digestion of calcium too. In this study, there were 15 genera in the rumen, 13 genera in the abomasum, three genera in the colon, and one genus in the cecum (Table 4) whose relative abundance were significantly different between the HC and LC groups. There were 13 bacteria groups in rumen, seven bacteria groups in abomasum, two bacteria groups in colon, and one in cecum (Figure 6), whose relative abundance were significantly associated with host TDC. These results indicated that the bacteria in the GIT played a vital role in influencing calcium digestibility. According to our knowledge, the present study was the first experiment evaluating the influence of the composition and structure of gastrointestinal bacteria on the host TDC. The mechanism of gastro-intestinal bacteria impacting calcium digestion has not yet been fully understood. We speculated that it may be related to enzymes secreted by gastro-intestinal bacteria, which can catalyze the decomposition of calcium compounds. A previous experiment found that the supplementation of phytase (myo-inositol hexakisphosphate phosphohydrolase) in swine diet significantly improved the digestion of Cafrom Canola meal [6]. Phytase presents in many plant-origin feed ingredients with high concentrate, such as canola meal. A molecule of phytic acid has six phosphate groups esterified to the inositol ring and can bind to six Ca^2+^ cations [32], and almost half of calcium in plant feed materials is bound to phytic acid [33], which causes the low utilization of calcium. Phytase are mainly produced by microorganisms, and ruminant GIT contains a large number of phytase-producing microorganisms such as Bifidobacterium [34], whose relative abundance of HC group was significantly higher than that of the LC group in abomasums in this study. It has been demonstrated that phytase are present in the gastrointestinal contents, and ruminants can utilize phytate calcium effectively without dietary phytase supplementation [35].

The results of the current study indicated that the bacteria influencing dietary calcium digestibility mainly came from the stomach rather than the intestine because the number of bacterial genera significantly associated with host TDC (28 in the stomach (rumen and abomasum) and only four in the intestine (jejunum, colon, and cecum)). It is well known that the microbial digestion mainly occurs in the rumen and chemical digestion in the jejunum of the ruminants. Therefore, the significant correlation between ruminal bacteria and dietary calcium digestibility are easy to understand. It was unexpected that a large number of bacteria in the abomasum were found to be significantly associated with host TDC in the present study. Abomasal 16S-DNA simply reflects the total influx of microbial matter from the rumen into the intestine rather than a digestive activity of microbes in this part of the GIT. Since pH is quite low in the abomasum, the microbial gene copies were derived from inactivated (dead) microbes. Hence, the abomasal gene copies are probably the best estimate for the overall composition of the rumen microbiota. An unexpected and hard to explain finding was that there were many bacteria in the GIT, which were significantly negatively related to the host TDC (Figure 6). The positive correlation between gastrointestinal bacteria and host TDC can be attributed to the existence of phytase-producing microbes; the negative correlation indicated that some bacteria can inhibit dietary calcium digestion. We speculated that the metabolite of these bacteria can catalyze the combination of free calcium with some substances in feed, such as protein and fat, which were not digestible. In addition, there might be a competitive or antagonistic relationship between these bacteria and phytase-producing microbes, and their large-scale reproduction can inhibit the growth of phytase-producing microorganisms, thereby reducing the production of phytase in the GIT.

This study found that some bacterial genera, even those located in different segments of the GIT, had the same correlation with TDC. For example, Ruminococcaceae_UCG-004 was significantly positively correlated with TDC in both the rumen and the abomasum, and Bacteroides in the rumen and the colon was negatively correlated with TDC. There was no bacterium exhibited opposite correlations with TDC when they were located in different parts of the GIT. These results indicated that the characteristics related to calcium digestion of bacteria were relatively stable.

## 5. Conclusions

In summary, there were noticeable individual variations in the TDC of goats. Obviously, the structure of the gastrointestinal microbiota of goats is related to the TDC. Additionally, rumen and abomasum microbes had the most enormous influence on host TDC compared with those of other gastrointestinal sections. Some gastro-intestinal bacteria, such as ruminal Prevotella, were beneficial for true host digestibility of dietary calcium.

## Figures and Tables

**Table 1:** The composition and nutritional ingredients of the experimental diet (dry matter basis).

Ingredients	Content (%)	
	Stage	
	I	II
Alfalfa meal	20.00	20.00
*Leymus chinensis*	35.00	35.00
Corn	38.47	39.15
Soybean meal	4.50	4.50
Premix ^1^	0.45	0.45
NaCl	0.45	0.45
Baking soda	0.45	0.45
CaCO_3_	0.08	-
CaHPO_4_	0.60	-
Total	100.00	100.00
Nutrition levels ^2^		
Metabolic energy (ME) (MJ/kg)	9.33	9.46
Crude protein (CP)	9.71	9.79
Acid detergent fiber (ADF)	24.07	24.07
Neutral detergent fiber (NDF)	36.11	36.20
Calcium (calcium)	0.52	0.35
Phosphorus (P)	0.33	0.22
Ca/P	1.58	1.59

Note: ^1^ Premix provides the following per kg of the diet: Fe: (as ferrous sulfate) 30 mg, Cu: (as copper sulfate) 10 mg, Zn: (as zinc sulfate) 50 mg, Mn: (as manganese sulfate) 60 mg, VA: 2 937 IU, VD: 343 IU, VE: 30 IU. ^2^ ME is a calculated value, and the others are measured values.

**Table 2:** Comparison of serum parameters, rumen fermentation parameters, and apparent digestibility of nutrients between-group HC and LC.

Items	HC (*n* = 8)	LC (*n* = 8)	*p*-Value
Serum parameters			
calcium (mmol/L)	2.35 ± 0.61	2.08 ± 0.50	0.230
P (mmol/L)	2.44 ± 0.61	2.33 ± 0.54	0.677
Alkaline phosphatase of blood (U/L)	204 ± 40.0	190 ± 71.8	0.644
Rumen fermentation parameters			
Rumen pH	6.35 ± 0.24	6.40 ± 0.17	0.622
Acetate (mmol/L)	30.8 ± 4.64	33.2 ± 6.52	0.416
Propionate (mmol/L)	10.4 ± 1.97	10.5 ± 1.09	0.925
Butyrate (mmol/L)	7.99 ± 1.12	7.80 ± 1.69	0.791
Acetate/Propionate	3.00 ± 0.48	3.18 ± 0.70	0.553
Apparent digestibility of nutrients (%)			
Dry matter (DM)	68.0 ± 1.96	69.0 ± 2.12	0.344
Ash	53.7 ± 3.65	56.2 ± 4.58	0.246
Crude Protein (CP)	45.6 ± 7.78	40.6 ± 12.4	0.352
Ether extract (EE)	67.8 ± 5.15	65.4 ± 4.68	0.345
Acid detergent fiber (ADF)	63.7 ± 3.01	62.9 ± 4.98	0.700
Neutral detergent fiber (NDF)	68.2 ± 2.13	70.1 ± 2.69	0.130

Note: HC: High Digestibility of Calcium group, LC: Low Digestibility of Calcium group.

**Table 3:** Comparison of the alpha diversity indices between the HC and LC groups (Mean ± SD).

Indices	Region	HC (*n* = 8)	LC (*n* = 8)	*p*-Value
Observed_species	Rumen	1502.37 ± 62.77	1603.25 ± 177.37	0.152
	Abomasum	1607.12 ± 105.13	1471.75 ± 142.00	0.048
	Jejunum	1629.25 ± 269.41	1678.12 ± 272.07	0.723
	Colon	1775.25 ± 323.41	1673.50 ± 314.31	0.534
	Cecum	1738.85 ± 316.67	1866.62 ± 174.17	0.342
Shannon	Rumen	7.83 ± 0.49	7.69 ± 0.33	0.532
	Abomasum	7.72 ± 0.48	7.27 ± 0.72	0.169
	Jejunum	6.81 ± 0.99	6.95 ± 1.05	0.787
	Colon	8.26 ± 0.83	8.36 ± 0.33	0.761
	Cecum	7.61 ± 2.03	8.28 ± 0.52	0.380
Simpson	Rumen	0.98 ± 0.16	0.98 ± 0.09	0.883
	Abomasum	0.97 ± 0.25	0.96 ± 0.25	0.581
	Jejunum	0.94 ± 0.40	0.96 ± 0.35	0.551
	Colon	0.97 ± 0.42	0.99 ± 0.04	0.336
	Cecum	0.92 ± 0.19	0.98 ± 0.07	0.307
Chao1	Rumen	1677.67 ± 102.15	1822.44 ± 202.62	0.093
	Abomasum	1818.53 ± 106.48	1725.10 ± 147.06	0.168
	Jejunum	1848.95 ± 305.37	2003.67 ± 363.87	0.373
	Colon	1921.01 ± 368.73	1827.71 ± 341.88	0.608
	Cecum	1898.19 ± 308.03	2154.06 ± 232.38	0.090
ACE	Rumen	1698.15 ± 74.93	1840.62 ± 196.68	0.076
	Abomasum	1847.20 ± 124.26	1724.50 ± 140.05	0.085
	Jejunum	1897.47 ± 315.26	2028.63 ± 349.36	0.444
	Colon	1962.28 ± 396.48	1853.83 ± 373.81	0.582
	Cecum	1941.96 ± 315.68	2147.38 ± 214.70	0.160
Goods_Coverage	Rumen	0.99 ± 0.00	0.99 ± 0.00	0.101
	Abomasum	0.99 ± 0.00	0.99 ± 0.00	0.776
	Jejunum	0.99 ± 0.00	0.99 ± 0.02	0.365
	Colon	0.99 ± 0.00	0.99 ± 0.02	0.675
	Cecum	0.99 ± 0.00	0.99 ± 0.00	0.016
PD_Whole_Tree	Rumen	81.80 ± 2.31	85.90 ± 6.42	0.111
	Abomasum	88.44 ± 3.48	83.25 ± 5.66	0.045
	Jejunum	89.29 ± 11.97	106.42 ± 39.23	0.257
	Colon	84.08 ± 15.27	79.06 ± 15.33	0.523
	Cecum	83.83 ± 13.37	88.49 ± 7.17	0.407

Note: HC: High Digestibility of Calcium group, LC: Low Digestibility of Calcium group.

**Table 4:** Distribution of microbes whose relative abundance is significantly different between HC and LC groups from Phylum to the genus.

Level Region Taxa			Relative Abundance (%)		
			HC	LC	*p*-Value
Phylum	R	Lentisphaerae	0.441 ± 0.107	0.673 ± 0.251	0.031
	A	Chloroflexi	0.413 ± 0.329	0.134 ± 0.058	0.033
		Planctomycetes	0.136 ± 0.077	0.033 ± 0.021	0.003
	Co	Bacteroidetes	18.099 ± 4.901	24.224 ± 4.171	0.018
		Fibrobacteres	0.487 ± 0.411	1.060 ± 0.561	0.035
Class	R	Clostridia	26.996 ± 6.174	21.095 ± 0.1.724	0.021
	A	Negativicutes	1.721 ± 0.576	1.073 ± 0.587	0.043
		Anaerolineae	0.412 ± 0.329	0.129 ± 0.049	0.031
		Planctomycetacia	0.135 ± 0.076	0.033 ± 0.021	0.003
	Co	Bacteroidia	17.908 ± 4.723	24.118 ± 4.191	0.015
		Fibrobacteria	0.486 ± 0.411	1.060 ± 0.561	0.035
Order	R	Clostridiales	26.996 ± 6.174	21.095 ± 1.723	0.021
		Saccharibacteria	0.136 ± 0.072	0.048 ± 0.046	0.011
	A	Selenomonadales	1.721 ± 0.575	1.074 ± 0.587	0.043
		Anaerolineales	0.412 ± 0.329	0.129 ± 0.049	0.031
		Planctomycetales	0.135 ± 0.077	0.033 ± 0.021	0.003
	Co	Bacteroidales	17.908 ± 4.723	24.118 ± 4.191	0.015
		Fibrobacterales	0.486 ± 0.411	1.060 ± 0.561	0.035
Family	R	Ruminococcaceae	10.051 ± 2.102	7.738 ± 1.551	0.025
		Prevotellaceae	18.255 ± 3.445	25.758 ± 4.885	0.003
		Christensenellaceae	2.132 ± 0.630	1.314 ± 0.386	0.007
		Bacteroidaceae	0.081 ± 0.068	0.207 ± 0.145	0.044
		Saccharibacteria	0.136 ± 0.072	0.048 ± 0.046	0.011
		Family_XIII	0.995 ± 0.263	0.691 ± 0.123	0.011
	A	Rikenellaceae	6.706 ± 1.832	4.409 ± 2.041	0.033
		Acidaminococcaceae	1.354 ± 0.446	0.820 ± 0.492	0.039
		Anaerolineaceae	0.412 ± 0.329	0.129 ± 0.049	0.031
		Planctomycetaceae	0.135 ± 0.076	0.033 ± 0.021	0.003
	Co	Ruminococcaceae	4.125 ± 0.990	5.680 ± 1.287	0.017
		Peptostreptococcaceae	0.486 ± 0.411	1.060 ± 0.561	0.035
		Acidaminococcaceae	0.315 ± 0.117	0.593 ± 0.339	0.046
Genus	R	Prevotella_1	13.459 ± 3.370	18.619 ± 3.513	0.010
		*Prevotellaceae_UCG-003*	1.197 ± 0.256	1.718 ± 0.563	0.032
		*Christensenellaceae_R-7_group*	2.071 ± 0.624	1.276 ± 0.384	0.008
		*Pseudobutyrivibrio*	0.725 ± 0.252	1.833 ± 1.353	0.039
		*Prevotellaceae_UCG-004*	0.225 ± 0.111	0.411 ± 0.145	0.012
		*Ruminococcus_2*	0.612 ± 0.029	0.204 ± 0.069	0.002
		*Quinella*	0.692 ± 0.571	0.213 ± 0.187	0.041
		*Succinivibrionaceae_UCG-002*	0.174 ± 0.187	0.539 ± 0.403	0.036
		*Ruminococcaceae_UCG-004*	0.218 ± 0.070	0.136 ± 0.054	0.021
		*Bacteroides*	0.081 ± 0.068	0.207 ± 0.145	0.044
		*Mogibacterium*	0.120 ± 0.033	0.065 ± 0.025	0.002
		*Family_XIII_UCG-002*	0.117 ± 0.038	0.061 ± 0.029	0.005
		*Candidatus_Saccharimonas*	0.136 ± 0.072	0.048 ± 0.046	0.011
		*Ruminococcaceae_UCG-007*	0.100 ± 0.041	0.038 ± 0.027	0.003
	A	*Rikenellaceae_RC9_gut_group*	6.191 ± 1.676	4.075 ± 1.951	0.035
		*Succiniclasticum*	1.351 ± 0.449	0.820 ± 0.492	0.041
		*Ruminococcus_1*	1.351 ± 0.449	0.820 ± 0.492	0.049
		*Succinivibrio*	0.979 ± 0.265	0.649 ± 0.342	0.031
		*Lachnospiraceae_NK3A20_group*	0.294 ± 0.172	0.804 ± 0.576	0.012
		*Ruminococcaceae_UCG-014*	0.550 ± 0.179	0.761 ± 0.101	0.012
		*Lachnospiraceae_ND3007_group*	0.627 ± 0.232	0.361 ± 0.116	0.038
		*Ruminococcaceae_UCG-010*	0.658 ± 0.239	0.388 ± 0.230	0.026
		*Eubacterium_coprostanoligenes_group*	0.536 ± 0.179	0.276 ± 0.141	0.006
		*Acetitomaculum*	0.123 ± 0.066	0.218 ± 0.104	0.048
		*Ruminococcaceae_UCG-004*	0.199 ± 0.064	0.117 ± 0.070	0.029
		*p-1088-a5_gut_group*	0.108 ± 0.066	0.031 ± 0.021	0.007
		*Prevotellaceae_YAB2003_group*	0.037 ± 0.022	0.117 ± 0.088	0.026
	Co	*Bacteroides*	4.125 ± 0.990	5.680 ± 1.287	0.017
		*Fibrobacter*	0.486 ± 0.410	1.059 ± 0.559	0.035
		*Ruminiclostridium_1*	0.161 ± 0.087	0.296 ± 0.135	0.033
	Ce	*Butyrivibrio*	0.058 ± 0.015	0.125 ± 0.055	0.008

Note: HC: High Digestibility of Calcium group, LC: Low Digestibility of Calcium group.
